# A study on factors influencing Chinese undergraduate EFL learners’ self-directed use of mobile English learning resources

**DOI:** 10.3389/fpsyg.2023.1189055

**Published:** 2023-10-10

**Authors:** Fengdan Shen, Linling Liang, Yufang Feng

**Affiliations:** ^1^Guangling College, Yangzhou University, Yangzhou, Jiangsu, China; ^2^Nagoya University, Nagoya, Japan; ^3^College of International Studies, Yangzhou University, Yangzhou, Jiangsu, China

**Keywords:** mobile English learning resources, mobile-assisted language learning, Chinese university students, unified theory of acceptance and use of technology, structural equation modeling

## Abstract

This study aimed to examine the factors that potentially impact the self-directed use of mobile English learning resources (MELR). The participants were 206 Chinese undergraduate EFL learners at Yangzhou University in Mainland China. Applying and modifying the Unified Theory of Acceptance and Use of Technology (UTAUT), this study involved six constructs, including students’ performance expectancy, effort expectancy, social influence, facilitating conditions, perceived playfulness, and behavioral intention to use MELR. The structural equation modeling (SEM) technique was adopted to analyze the data collected from the questionnaire. The findings showed that facilitating conditions acted as the most significant predictor of behavioral intention to adopt MELR, followed by effort expectancy, perceived playfulness, and performance expectancy. However, social influence did not have significant effects on students’ use of MELR. Pedagogical implications for teachers and students were also presented in the end.

## Introduction

With the increasing ownership of mobile devices, mobile technologies are widely adopted in foreign language learning. Students are provided with flexible access to learning resources of all types anywhere and at any time (Kukulska-Hulme, 2009). Mobile devices are not only used in formal education context but also in informal settings (Chen, 2013). As touch-screen smartphones are available to many Chinese university students, they have applied various mobile-assisted language learning (MALL) platforms in their self-directed language learning (Yu et al., 2019). As a result, mobile English learning resources (MELR) are becoming increasingly popular among university English learners in China. For example, Chinese students use various mobile APPs to improve their English proficiency and communication skills, such as Youdao Dictionary, Baicizhan, Shanbay Words, Coursera, and TED (Zhang and Pérez-Paredes, 2019). As students’ intentions toward mobile technology will influence their actual use of mobile English learning, it is essential to explore the factors influencing students’ acceptance and usage of MELR so as to support informal and self-directed English learning in China.

The present study aimed to examine the factors that affect Chinese undergraduate students’ intention to adopt MELR. The current study developed a research model of MELR acceptance mainly based on the Unified Theory of Acceptance and Use of Technology model (UTAUT) as well as prior researches. The constructs examined in the research model were students’ performance expectancy, effort expectancy, social influence, facilitating conditions, perceived playfulness, and behavioral intention to adopt MELR.

## Literature review

### Mobile English learning resources

Previous researchers have conducted some studies on Chinese university students’ acceptance and use of MELR. In a study that examined Chinese undergraduates’ perceptions of mobile English learning, Zou and Yan (2014) found that 78% of the students had a positive attitude toward using MELR. In addition, Zhang and Pérez-Paredes (2019) reported that passing exams and improving test scores were the most important reasons for students to use MELR. This is in line with Xue’s (2014) finding that using mobile devices in English learning was helpful to improve students’ test scores. As for the most frequently used MELR, vocabulary and translation applications were far more popular than other listening, speaking, reading, and writing resources (Lai et al., 2022). This is consistent with Steel’s (2012) finding that vocabulary acquisition was the most popular learning activity among Chinese language learners when it comes to MELR. In order to explore the effectiveness of MELR on English language learning, Yin (2013) suggested that Wechat – a social media platform, was an effective way to prepare students for CET-4 and CET-6 test through enhancing vocabulary, grammar, etc. Ruan and Ma (2014) also demonstrated that a designed mobile APP could facilitate students’ grammar learning.

Numerous research studies have been carried out to investigate the factors that influenced students’ self-directed use of MELR. García Botero et al. (2018) extended the UTAUT model to explore the factors that affected students’ behavioral intentions toward using mobile devices for English learning. Their findings indicated that social influence, performance expectancy, and facilitating conditions impacted students’ attitudes to mobile English learning, and behavioral intentions had effects on actual use of mobile English learning. Similarly, Hoi (2020) surveyed higher education learners in Vietnam to understand their adoption of mobile-assisted language learning (MALL). They found that attitude and performance expectancy had significant effects on learners’ behavioral intentions toward using mobile-assisted language learning, while facilitating conditions exerted no direct influence on learners’ MALL acceptance. In terms of English vocabulary learning, Guo and Li (2022) suggested that performance expectancy, effort expectancy, price value, facilitation condition, and habit had a significant positive impact on the use of English vocabulary APP learning by college students. Using a different model (TAM), Kim and Lee (2016) investigated Korean students’ adoption of mobile-assisted language learning and potential factors affecting their MALL use. Results indicated that perceived ease of use, perceived usefulness, perceived enjoyment, and content reliability significantly predicted students’ acceptance of mobile English learning. However, few studies have been conducted to explore factors influencing Chinese students’ use of mobile devices, especially in higher education context. To fill in this gap, this study aims to examine the factors that affect Chinese university students’ self-directed use of mobile English learning resources applying the UTAUT model.

### Theocratical background

The Unified Theory of Acceptance and Use of Technology (UTAUT) was developed by Venkatesh et al. (2003), which is also the most recent model to explain the users’ technology acceptance process. The UTAUT model is applicable in studies exploring technology acceptance among college students (Kumar and Bervell, 2019), which has also been one of the most popular models for mobile learning (Venkataraman and Ramasamy, 2018). The UTAUT has four core constructs, including performance expectancy, effort expectancy, social influence, and facilitating conditions. Their definitions are summarized as follows (Venkatesh et al., 2003):

- Performance Expectancy (PE): This refers to “the degree to which an individual believes that using the system will help him or her to attain gains in job performance.”
- Effort Expectancy (EE): This refers to “the degree of ease associated with the use of the system.”
- Social Influence (SI): This refers to “the degree to which an individual perceives that important others believe he or she should use the new system.”
- Facilitating Conditions (FC): This refers to “the degree to which an individual believes that an organizational and technical infrastructure exists to support the use of the system.”

**Figure 1:**
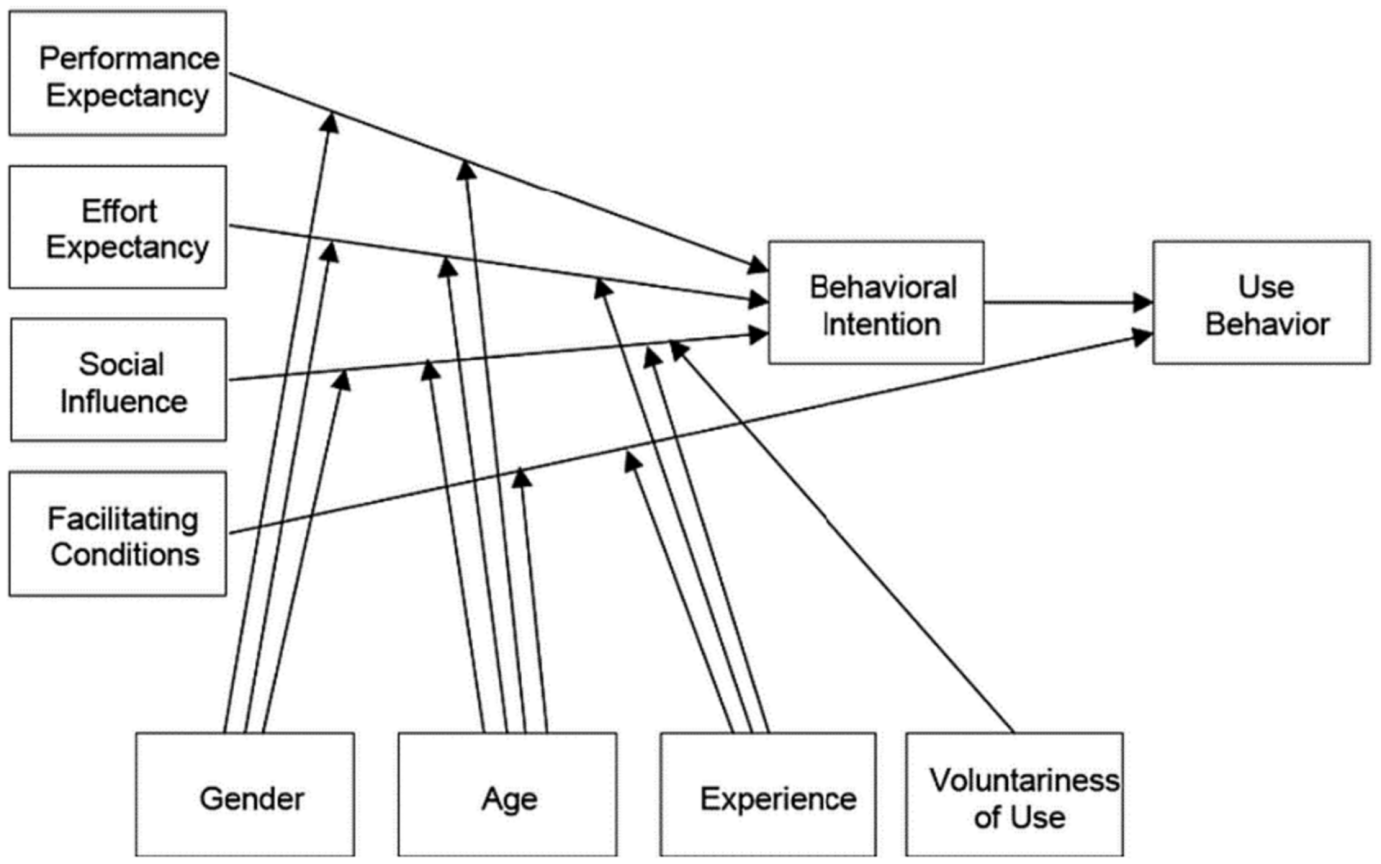
Unified theory of acceptance and use of technology (UTAUT).

As shown in the UTAUT model (Figure 1), performance expectancy, effort expectancy, and social influences have an impact on behavioral intention while facilitating conditions have an impact on use behavior. In addition, gender, age, experience, and voluntariness have a moderating effect on behavioral intention.

### Research model and hypotheses

**Figure 2:**
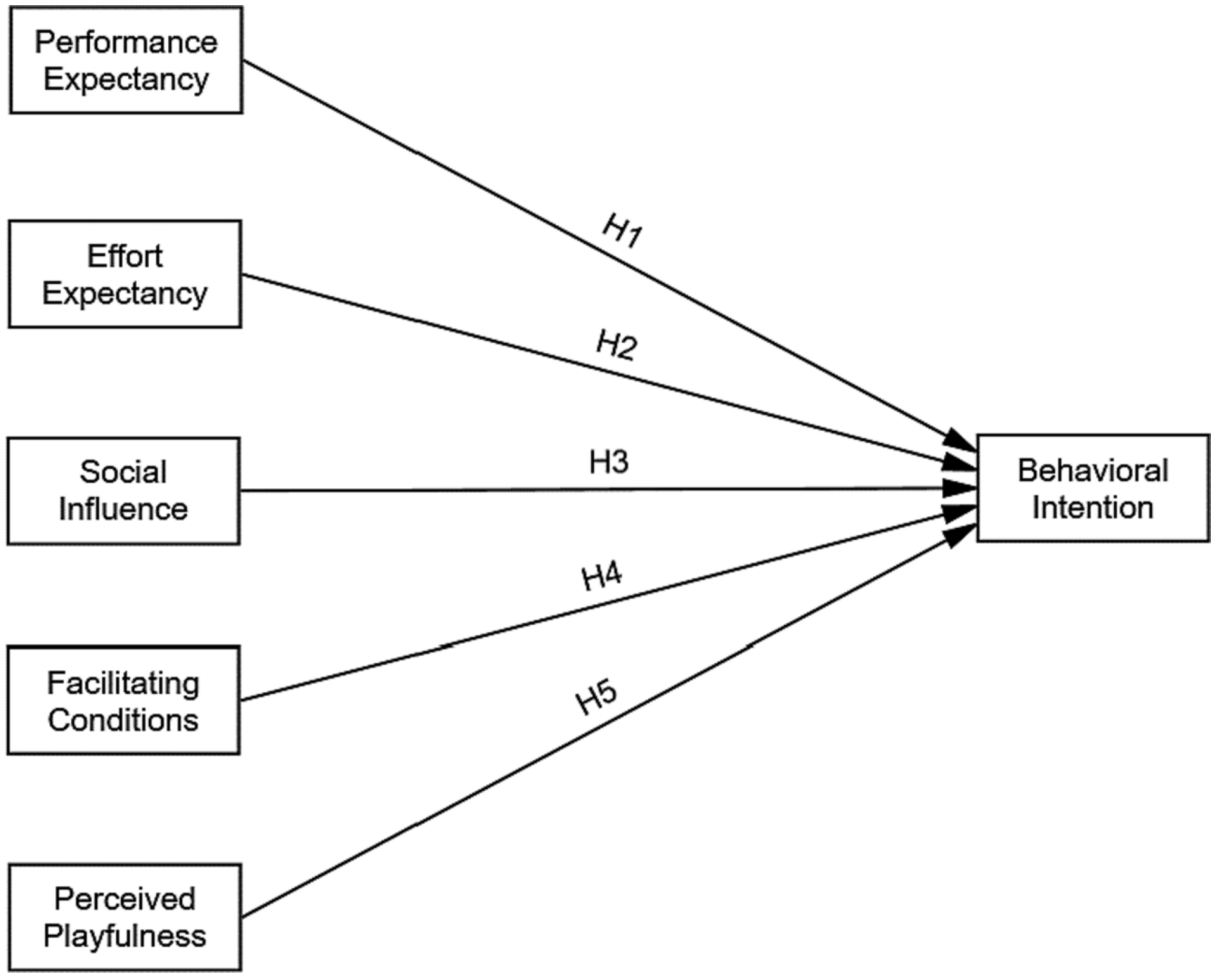
Research model.

The objective of this study is to investigate the factors that affect behavioral intention toward adopting MELR among Chinese undergraduate EFL learners. In this model, Mobile English learning resources (MELR) refer to mobile applications, English podcasts, online courses, e-books, and websites delivered via mobile devices. Based on the UTAUT, the author proposed a research model (Figure 2) and made some modifications. First, the proposed model has added perceived playfulness (PP) as an independent construct. Perceived playfulness has been defined as “level of a user’s concentration, curiosity and enjoyment in the interaction with the technology environment” (Moon and Kim, 2001), which has been found to predict intention to adopt mobile learning (Wang et al., 2009). Second, this study only investigated the main hypotheses while dropping the effects of the moderators. Third, this study eliminated the construct of use behavior and only measured behavioral intention. Thus, this study has formulated and tested the following five hypotheses:

*Hypothesis 1*: Performance expectancy has a significant and positive effect on behavioral intention to use MELR.

*Hypothesis 2*: Effort expectancy has a significant and positive effect on behavioral intention to use MELR.

*Hypothesis 3*: Social influence has a significant and positive effect on behavioral intention to use MELR.

*Hypothesis 4*: Facilitating conditions have a significant and positive effect on behavioral intention to use MELR.

*Hypothesis 5*: Perceived playfulness has a significant and positive effect on behavioral intention to use MELR.

## Methods

### Participants

This study consisted of 206 Chinese undergraduate students with English education majors at Yangzhou University in Mainland China. Specifically, 21 participants (10.2%) were male, and 185 (89.8%) were female. Participants were distributed almost equally across four different levels. 46 participants (22.3%) were Year one; 58 (28.2%) were Year two; 52 (25.2%) were Year three; 50 (24.3%) were Year four. This study used online survey called Sojump to collect data. An anonymous link was administered via social media platforms such as QQ and WeChat. A total of 247 questionnaires were returned and 206 were considered valid for further analysis.

### Instrument

This study used a quantitative questionnaire to collect data, which consisted of two parts. The first part collected demographic information, including gender, age, and grade. The second part involved 18 UTAUT-based items to measure six constructs, namely performance expectancy, effort expectancy, social influence, facilitating conditions, perceived playfulness, and behavioral intention. All items were adapted from previous studies (Wang et al., 2009; Chung et al., 2015; Fagan, 2019; Hoi, 2020; Nikolopoulou et al., 2020). The items were scored on a five-point Likert scale, ranging from strongly disagree to strongly agree. The items and their sources are listed in the Appendix. The questionnaire was translated from English to Chinese so that participants can better understand the items.

### Data analysis

In this study, Structural Equation Modeling (SEM) with Amos 26 tool was used to analyze the data. This study followed the two-step approach recommended by Anderson and Gerbing (1988).

The first step was to assess the validity and reliability of the measurement model. In this step, factor loadings, composite reliability (CR), average variance extracted (AVE), Cronbach’s alpha, and inter-construct correlations were calculated to describe the validity and reliability of each construct (Fornell and Larcker, 1981).

The second step was to analyze the structural model to test the research hypotheses. To achieve a good model fit, Chi-square to its degree of freedom (CMIN/df) should be lower than 3 (Schumacker and Lomax, 2010). Goodness-of-fit index (GFI) and adjusted goodness-of-fit index (AGFI) should be greater than 0.8 (Forza and Filippini, 1998). Comparative fit index (CFI) and Tucker-Lewis index (TLI) should be above 0.9 (Kline, 2005). Root mean square error of approximation (RMSEA) and standardized root mean square residual (SRMR) should be lower than 0.08 (Steiger, 2007).

## Results

### Measurement model

Confirmatory factor analysis (CFA) was conducted to examine the convergent and discriminant validity of the measurement model. Convergent validity was measured by factor loadings, composite reliability (CR), and average variance extracted (AVE). The recommended values for factor loadings, CR, and AVE were 0.6, 0.7, and 0.5, respectively (Hair, 2006; Awang, 2015). As shown in Table 1, all factor loadings were above 0.6. All CRs were higher than 0.7 and all AVEs were above 0.5, indicating that all constructs had appropriate convergent validity. In addition, all Cronbach’s Alpha values were larger than 0.70, indicating that all constructs had acceptable internal consistency.

**Table 1:** Reliability and convergent validity.

Construct	Items	Factor loading	Item reliability	CR	AVE	Cronbach’s alpha
PE	PE1	0.653	0.426	0.785	0.551	0.783
	PE2	0.732	0.536			
	PE3	0.832	0.692			
EE	EE1	0.860	0.740	0.876	0.701	0.876
	EE2	0.803	0.645			
	EE3	0.848	0.719			
SI	SI1	0.661	0.437	0.760	0.519	0.754
	SI2	0.862	0.743			
	SI3	0.614	0.377			
FC	FC1	0.807	0.651	0.843	0.642	0.833
	FC2	0.861	0.741			
	FC3	0.731	0.534			
PP	PP1	0.938	0.880	0.942	0.845	0.940
	PP2	0.926	0.857			
	PP3	0.893	0.797			
BI	BI1	0.823	0.677	0.874	0.698	0.870
	BI2	0.870	0.757			
	BI3	0.813	0.661			

Discriminant validity would be regarded as appropriate if the square root of the AVE of each construct was greater than its correlation coefficients with other constructs (Gefen et al., 2000). As presented in Table 2, for each construct, the square root of AVE was greater than the correlation coefficients with other constructs, demonstrating good discriminant validity.

**Table 2:** Discriminant validity.

	PP	FC	SI	EE	PE	BI
PP	0.919					
FC	0.610	0.801				
SI	0.430	0.487	0.720			
EE	0.563	0.607	0.371	0.837		
PE	0.680	0.506	0.382	0.652	0.743	
BI	0.720	0.760	0.429	0.731	0.700	0.836

### Structural model

The second step was to test the structural model. The model fit was examined by several key goodness-of-fit indices, which were summarized in Table 3. Based on the results, the research model showed a good fit to the data.

**Table 3:** CFA statistics of model fit.

Model fit index	Criteria	Research model
CMIN /df	< 3	1.986
GFI	>0.8	0.886
AGFI	>0.8	0.838
CFI	>0.9	0.951
TLI	>0.9	0.937
RMSEA	<0.08	0.069
SRMR	<0.08	0.051

**Figure 3:**
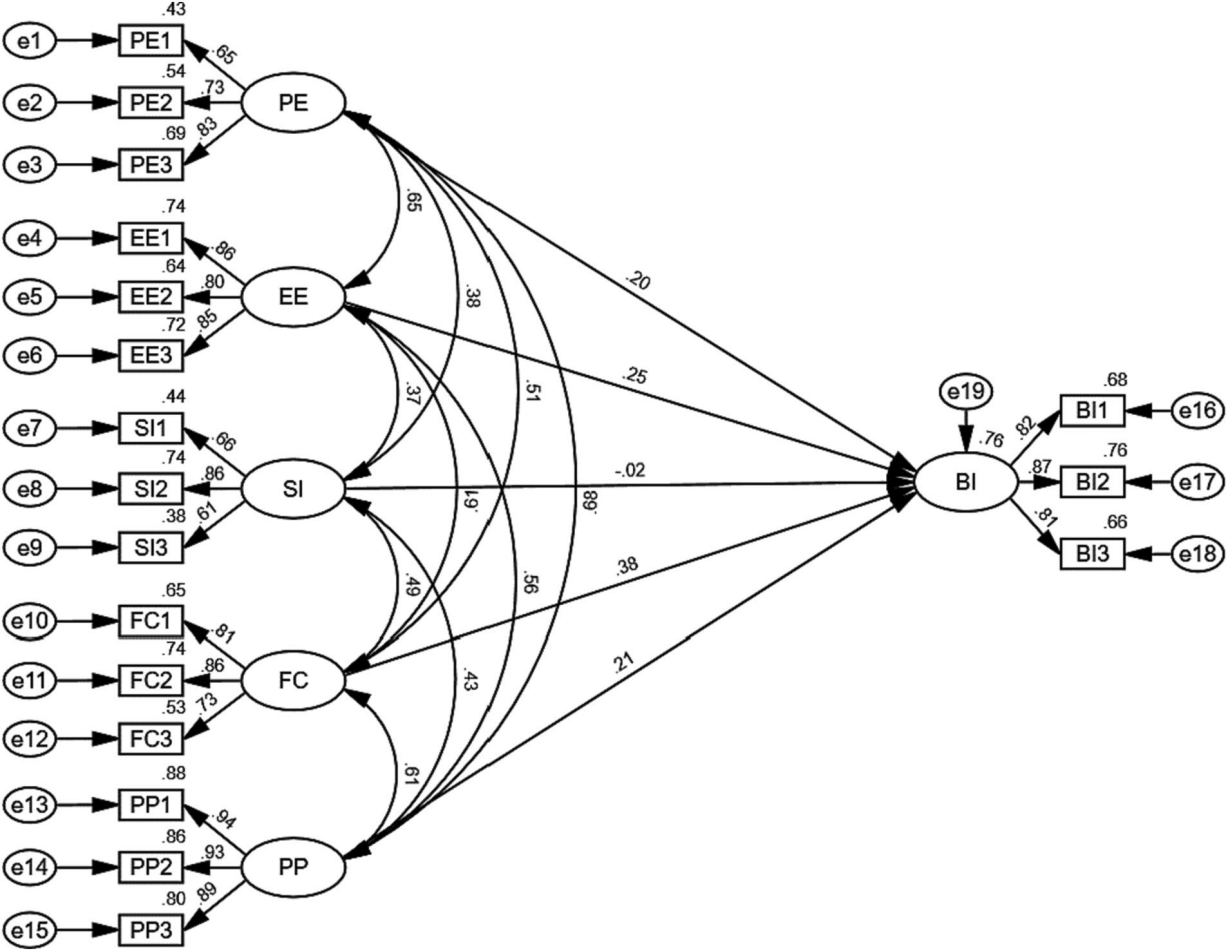
Structural model results.

As shown in Figure 3, the model explained 76% of the variance in behavioral intention, which was higher than the threshold of 70% recommended by Venkatesh et al. (2003). Table 4 also listed the path coefficients and their significance level for each hypothesis. Among all the five hypotheses, four hypotheses were supported. No significant relationship was found between social influence and behavioral intention (β = −0.021, *p* > 0.05). Thus, Hypothesis 3 was not supported. In addition, facilitating conditions (β = 0.383, *p* < 0.001) exerted the greatest influence on behavioral intention, followed by effort expectancy (β = 0.253, *p* < 0.01), perceived playfulness (β = 0.214, *p* < 0.01), and performance expectancy (β = 0.205, *p* < 0.05). Therefore, Hypothesis 1, Hypothesis 2, Hypothesis 4, and Hypothesis 5 were supported.

**Table 4:** Path analysis results.

Hypothesis	Standardized path coefficient	T value	*p* value	Result
H1: PE → BI	0.205	2.233	0.026*	Supported
H2: EE → BI	0.253	3.110	0.002**	Supported
H3: SI → BI	−0.021	−0.338	0.736	Not supported
H4: FC → BI	0.383	4.636	0.000***	Supported
H5: PP → BI	0.214	2.690	0.007**	Supported

*p < 0.05, **p < 0.01, ***p < 0. 001.

## Discussion

The aim of this study was to identify the factors in the UTAUT that affected students’ self-directed use of MELR in Chinese higher education context. Based on the results, the UTAUT model can explain 76% of students’ behavioral intention to adopt MELR for self-directed English language learning. All except one of the relationships proposed in the UTAUT were found to be significant.

### Facilitating conditions

Facilitating conditions were the most powerful determinant of behavioral intention to adopt MELR. This finding was in accordance with the results of previous studies on mobile English learning (Mekhzoumi et al., 2018; Ahmed et al., 2021; Guo and Li, 2022) and other studies on mobile learning in general (Al-Adwan et al., 2018; Shameem and Sanjeetha, 2021), which consolidates the importance of this construct in the UTAUT model. When students perceive that necessary resources and technical support are available to them, they will have stronger intentions to use MELR for English learning. However, this finding was contrary to Hoi, 2020 study in Vietnam, where facilitating conditions were shown to have no direct effect on the adoption of mobile assisted language learning. One possible cause could be that students involved in this study might have more abundant MELR resources and better technical support, such as high-speed internet access. This would encourage Chinese undergraduate students to adopt mobile devices for English language learning.

### Effort expectancy

Effort expectancy was the second most important predictor of behavioral intention toward using MELR. This is consistent with other findings in mobile English learning context (Mekhzoumi et al., 2018; Guo and Li, 2022) and in mobile learning in general (Nassuora, 2013; Fagan, 2019). This finding implies that students who perceive MELR easy to use will have more positive intentions to adopt MELR for English learning. This result was also demonstrated by Kim and Lee, 2016 research. Using a different model (TAM), they found that effort expectancy was an important factor that affected the usage of mobile assisted language learning among Korean students. Therefore, when developing mobile English learning applications, designers should take this factor into consideration, making applications as user-friendly as possible.

### Perceived playfulness

Perceived playfulness was also a significant indicator of MELR adoption. This result aligned with Doan’s (2018) research on mobile English learning and other studies on mobile learning in general (Jawad and Hassan, 2015; Karimi, 2016). As expected, when MELR are perceived as pleasurable and enjoyable, students will be more likely to intend to use mobile devices for English learning. A potential reason for this result is that mobile English learning in China mainly occurs in informal settings outside class (Chen, 2013). Consequently, students will expect mobile English learning platforms to be interesting to use. Thus, these platforms should strive to create enjoy-and-learn environment so as to engage students.

### Performance expectancy

Finally, performance expectancy had a positive influence on students’ behavioral intention toward adopting MELR. This result coincided with other studies on the effect of performance expectancy on behavioral intention (Fagan, 2019; Nikolopoulou et al., 2020). Some studies also suggested that performance expectancy was the most important predictor of students’ intention to adopt mobile English learning (Mekhzoumi et al., 2018; Hoi, 2020). However, this study found that performance expectancy exerted the least influence on behavioral intention compared to other constructs. There are two possible explanations for such difference. First, Chinese students mainly regard MELR as supplementary English language learning material in that they adhere to traditional textbooks (Zhang and Pérez-Paredes, 2019). This will reduce their expectation of improving academic performance through MELR. Another reason involves the limitations of MELR. Small screen size, typing difficulties, and poor application design further undermine students’ desire to improve language performance using MELR.

### Social influence

Notably, social influence did not have a significant effect on behavioral intention to adopt MELR. This is in line with the findings of Arain et al. (2019), Fagan (2019), and Abbad (2021), and contrary to the findings of Ameri et al. (2020) and Ahmed et al. (2021). A possible explanation is that students examined in this study belong to digital natives who grow up in digital environment. When it comes to MELR, they are more comfortable with self-directed learning and thus are less influenced by instructors or peers.

## Conclusion

This study has explored the factors that predict Chinese undergraduate EFL learners’ use of MELR. The major findings are summarized as follows. Social influence was not significantly associated with behavioral intention. Facilitating conditions were the most powerful determinant of behavioral intention to use MELR. Effort expectancy, perceived playfulness, and performance expectancy also had a positive effect on students’ behavioral intention to adopt MELR.

Based on the findings, this study provides some pedagogical implications for language teachers and APP developers to promote MELR for educational purposes. First, given that facilitation conditions act as the most important determinant, it is important to provide proper assistance to students who have difficulties using mobile devices for language learning. This will help to remove the obstacles that students may encounter when using MELR, thus increasing student’s learning efficiency. Second, the content of MELR should be as accessible and legible as possible so that students will be more likely to perceive MELR to be easy and useful. Students should be encouraged to provide immediate feedback, which is a great way to improve usage experience. Third, MELR materials should be designed to be interesting and engaging. Quizzes and games can be installed to attract more students to use MELR. Finally, in order to improve the productivity and effectiveness of MELR, it is important to ensure the content quality of MELR materials so that students can improve their learning outcomes with the use of MELR.

### Limitations and suggestions for future research

This study has some limitations. First, this study had a relatively limited sample size with only 206 students at Yangzhou University. Thus, future research can involve more students and be conducted in other universities in order to generalize the results. Second, it only analyzed the core constructs of the UTAUT model and did not include the effects of potential moderators (age, gender, experience, and voluntariness) on the main relationships. Future studies can include these moderators to have a better understanding of students’ behavior intention. Finally, this study only investigated behavioral intention with a self-reported measure, which may change over time due to new knowledge and experience. In order to gain more accurate results, it is helpful to use a longitudinal study to measure students’ actual use of MELR.

## Data availability statement

The original contributions presented in the study are included in the article/supplementary material, further inquiries can be directed to the corresponding author.

## Author contributions

All authors listed have made a substantial, direct, and intellectual contribution to the work and approved it for publication.

## Appendix

**Table:**

Constructs	Items
Performance expectancy (PE)	PE1: I find it useful to adopt MELR for English learning.
	PE2: Using MELR for English learning increases my productivity.
	PE3: Using MELR for English learning improves my grades.
Effort expectancy (EE)	EE1: It is easy for me to become skillful at using MELR for English learning.
	EE2: I find it easy to use MELR for English learning.
	EE3: Using MELR for English learning is easy for me to adapt to.
Social influence (SI)	SI1: My friends’ recommendation influences my decision to use MELR for English learning.
	SI2: I use MELR for English learning if my instructors recommend them.
	SI3: My parents are supportive of the use of MELR for English learning.
Facilitating conditions (FC)	FC1: I have the resources necessary to use MELR for English learning.
	FC2: I have the knowledge necessary to use MELR for English learning.
	FC3: Using MELR to learn English is compatible with other technologies I use.
Perceived playfulness (PP)	PP1: Using MELR to learn English gives me enjoyment.
	PP2: Using MELR to learn English stimulates my curiosity.
	PP3: Using MELR to learn English leads to my exploration.
Behavioral intention (BI)	BI1: I would continue to use MELR for English learning.
	BI2: I would use MELR frequently for English learning.
	BI3: I would strongly recommend others to use MELR for English learning.

## References

[ref1] Abbad M. M. M. (2021). Using the UTAUT model to understand students’ usage of e-learning systems in developing countries. Educ. Inf. Technol. 26, 7205–7224. doi: 10.1007/s10639-021-10573-5, PMID: 34025204 PMC8122219

[ref2] Ahmed R. R. Štreimikienė D. Štreimikis J. (2021). The extended UTAUT model and learning MANAGEMENT system during COVID-19: evidence from PLS-SEM and conditional process modeling. J. Bus. Econ. Manag. 23, 82–104. doi: 10.3846/jbem.2021.15664

[ref3] Al-Adwan A. S. Al-Madadha A. Zvirzdinaite Z. (2018). Modeling students’ readiness to adopt Mobile learning in higher education: an empirical study. Int. Rev. Res. Open Dist. Learn. 19, 221–241. doi: 10.19173/irrodl.v19i1.3256

[ref4] Ameri A. Khajouei R. Ameri A. Jahani Y. (2020). Acceptance of a mobile-based educational application (LabSafety) by pharmacy students: an application of the UTAUT2 model. Educ. Inf. Technol. 25, 419–435. doi: 10.1007/s10639-019-09965-5

[ref5] Anderson J. C. Gerbing D. W. (1988). Structural equation modeling in practice: a review and recommended two-step approach. Psychol. Bull. 103, 411–423. doi: 10.1037/0033-2909.103.3.411

[ref6] Arain A. A. Hussain Z. Rizvi W. H. Vighio M. S. (2019). Extending UTAUT2 toward acceptance of mobile learning in the context of higher education. Univ. Access Inf. Soc. 18, 659–673. doi: 10.1007/s10209-019-00685-8

[ref7] Awang Z. (2015). SEM made simple: A gentle approach to learning Sructural equation modelling. Selangor, Malaysia: MPWS Rich Publication.

[ref8] Chen X.-B. (2013). Tablets for informal language learning: student usage and attitudes. Lang. Learn. Technol. 17, 20–36.

[ref9] Chung H.-H. Chen S.-C. Kuo M.-H. (2015). A study of EFL college students’ acceptance of Mobile learning. Procedia Soc. Behav. Sci. 176, 333–339. doi: 10.1016/j.sbspro.2015.01.479

[ref10] Doan N. T. T. (2018). Influences on smartphone adoption by language learners. Available at: https://www.semanticscholar.org/paper/Influences-on-smartphone-adoption-by-language-Doan/0c691de19cd61fdde94cef639008abca88185ee2

[ref11] Fagan M. H. (2019). Factors influencing student acceptance of Mobile learning in higher education. Comput. Sch. 36, 105–121. doi: 10.1080/07380569.2019.1603051

[ref12] Fornell C. Larcker D. F. (1981). Evaluating structural equation models with unobservable variables and measurement error. J. Mark. Res. 18:39. doi: 10.2307/3151312

[ref13] Forza C. Filippini R. (1998). TQM impact on quality conformance and customer satisfaction: a causal model. Int. J. Prod. Econ. 55, 1–20. doi: 10.1016/S0925-5273(98)00007-3

[ref14] García Botero G. Questier F. Cincinnato S. He T. Zhu C. (2018). Acceptance and usage of mobile assisted language learning by higher education students. J. Comput. High. Educ. 30, 426–451. doi: 10.1007/s12528-018-9177-1

[ref15] Gefen D. Straub D. Boudreau M.-C. (2000). Structural equation modeling and regression: guidelines for research practice. Commun. Assoc. Inf. Syst. 4, 1–76. doi: 10.17705/1CAIS.00407

[ref16] Guo H. Li Z. (2022). An analysis of the learning effects and differences of college students using English vocabulary APP. Sustain. For. 14:9240. doi: 10.3390/su14159240

[ref17] Hair J. F. (2006). Multivariate Data Analysis. New Jersey: Pearson Prentice Hall.

[ref18] Hoi V. N. (2020). Understanding higher education learners’ acceptance and use of mobile devices for language learning: a Rasch-based path modeling approach. Comput. Educ. 146:103761. doi: 10.1016/j.compedu.2019.103761

[ref19] Jawad H. H. M. Hassan Z. (2015). Applying Utaut to evaluate the acceptance of Mobile learning in higher education in Iraq. Available at: https://www.semanticscholar.org/paper/Applying-Utaut-to-Evaluate-the-Acceptance-of-Mobile-Jawad-Hassan/334857cd981b59d37362ba93bec49b2169bcaa1c

[ref20] Karimi S. (2016). Do learners’ characteristics matter? An exploration of mobile-learning adoption in self-directed learning. Comput. Hum. Behav. 63, 769–776. doi: 10.1016/j.chb.2016.06.014

[ref21] Kim G. Lee S. (2016). Korean students’ intentions to use Mobile-assisted language learning: applying the technology acceptance model. Int. J. Conten. 12, 47–53. doi: 10.5392/IJoC.2016.12.3.047

[ref22] Kline R. B. (2005). Principles and practice of structural equation modeling, 2nd ed (pp. 366). New York: Guilford Press.

[ref23] Kukulska-Hulme A. (2009). Will mobile learning change language learning? ReCALL 21, 157–165. doi: 10.1017/S0958344009000202

[ref24] Kumar J. A. Bervell B. (2019). Google classroom for mobile learning in higher education: modelling the initial perceptions of students. Educ. Inf. Technol. 24, 1793–1817. doi: 10.1007/s10639-018-09858-z

[ref25] Lai Y. Saab N. Admiraal W. (2022). University students’ use of mobile technology in self-directed language learning: using the integrative model of behavior prediction. Comput. Educ. 179:104413. doi: 10.1016/j.compedu.2021.104413

[ref26] Mekhzoumi O. Hamzah M. H. Krishnasamy H. N. (2018). Determinants of Mobile applications acceptance for English language learning in Universiti Utara Malaysia. Available at: https://www.semanticscholar.org/paper/Determinants-of-Mobile-Applications-Acceptance-for-Mekhzoumi-Hamzah/7c8981ddbc73504c8a9b46e7efa99c0aaa6a9adc

[ref27] Moon J.-W. Kim Y.-G. (2001). Extending the TAM for a world-wide-web context. Inf. Manag. 38, 217–230. doi: 10.1016/S0378-7206(00)00061-6

[ref28] Nassuora A. (2013). Students acceptance of Mobile learning for higher education in Saudi Arabia. Int. J. Learn. Manag. Syst. 1, 1–9. doi: 10.12785/ijlms/010101

[ref29] Nikolopoulou K. Gialamas V. Lavidas K. (2020). Acceptance of mobile phone by university students for their studies: an investigation applying UTAUT2 model. Educ. Inf. Technol. 25, 4139–4155. doi: 10.1007/s10639-020-10157-9

[ref30] Ruan Y. Ma Y. (2014). Intelligent mobile phone environment English mobile learning system design and implementation. Electronic Test 9, 13–15.

[ref31] Schumacker R. E. Lomax R. G. (2010). A beginner’s guide to structural equation modeling. (3rd edition). New York, NY: Routledge, Taylor & Francis Group.

[ref32] Shameem A. Sanjeetha M. B. F. (2021). M-learning systems usage: a perspective from students of higher educational institutions in Sri Lanka. J. Asian Finance Econ. Business 8, 637–645. doi: 10.13106/jafeb.2021.vol8.no8.0637

[ref33] Steel C. (2012). Fitting learning into life: Language students’ perspectives on benefits of using mobile apps. In Paper Presented at the Ascilite.

[ref34] Steiger J. H. (2007). Understanding the limitations of global fit assessment in structural equation modeling. Personal. Individ. Differ. 42, 893–898. doi: 10.1016/j.paid.2006.09.017

[ref35] Venkataraman J. B. Ramasamy S. (2018). Factors influencing mobile learning: a literature review of selected journal papers. Int. J. Mobile Learn. Organ. 12:99. doi: 10.1504/IJMLO.2018.090836

[ref36] Venkatesh V. Morris M. G. Davis G. B. Davis F. D. (2003). User acceptance of information technology: toward a unified view. MIS Q. 27, 425–478. doi: 10.2307/30036540

[ref37] Wang Y.-S. Wu M.-C. Wang H.-Y. (2009). Investigating the determinants and age and gender differences in the acceptance of mobile learning. Br. J. Educ. Technol. 40, 92–118. doi: 10.1111/j.1467-8535.2007.00809.x

[ref38] Xue J. (2014). Research on construction and development of m-learning mode of college English. Experim. Technol. Manag. 31, 176–179.

[ref39] Yin Y. (2013). Using WeChat to support undergraduates’ preparation for CET 4. Sci. Technol. Vision 25, 56–57.

[ref40] Yu Z. Zhu Y. Yang Z. Chen W. (2019). Student satisfaction, learning outcomes, and cognitive loads with a mobile learning platform. Comput. Assist. Lang. Learn. 32, 323–341. doi: 10.1080/09588221.2018.1517093

[ref41] Zhang D. Pérez-Paredes P. (2019). Chinese postgraduate EFL learners’ self-directed use of mobile English learning resources. Comput. Assist. Lang. Learn. 34, 1128–1153. doi: 10.1080/09588221.2019.1662455

[ref42] Zou B. Yan X. (2014). Chinese students’ perceptions of using Mobile devices for English learning. Int. J. Computer-Assisted Lang. Learn. Teach. 4, 1–14. doi: 10.4018/ijcallt.2014070102

